# 

**Published:** 1851-04

**Authors:** 


### CONTENTS

### OF NO. IV.

---

### ORIGINAL COMMUNICATIONS.

### ESSAYS AND CASES.

ART. I.—Epidemic Influences, and the duties of the Medical Profession in relation to them. By THEODORE S. BELL, M. D., of Louisville, Ky. - - - - - - - 277

ART. II.—Six cases of Phthisis Pulmonalis treated by Cod-Liver Oil. By DAVID W. YANDELL, M. D., of Louisville, Ky. Formerly attending Physician for the Faculty, at the Louisville Marine Hospital: One of the physicians to that Institution, etc. - - - - - 344

ART. III.—A Case of Urinary Calculus, attended with peculiar circumstances and treated by Lithotrity. By L. A. DUGAS, M. D., Prof. of Surgery in the Medical College of Georgia. - - - - - - - - - 359

### ORIGINAL INTELLIGENCE.

University of Louisville, - - - - - - - - 364

Meetings of Learned Bodies, - - - - - - - - 366

Dr. Warren's Address before the American Medical Association of Cincinnati, - - - - - - - - - - - 366

Proceedings of the Medical Association of the State of Alabama, begun and held in the city of Mobile, December, 1850, - 367

Nashville Journal of Medicine and Surgery, - - - - 368

The Stethoscope, and Virginia Medical Gazette, a monthly Journal of Medicine and the Collateral Sciences. Edited by P. Claiborne Gooch, M. D., - - - - - - - - 368

